# Runtime Firmware Update for 32-Bit Microcontrollers with Instruction Cache Under Concurrent Task Execution

**DOI:** 10.3390/s26185813

**Published:** 2026-09-14

**Authors:** Bernardino Pinto Neves, Victor D. N. Santos, José Eduardo G. Oliveira, António Valente

**Affiliations:** 1 Engineering Department, School of Sciences and Technology, University of Trás-os-Montes and Alto Douro (UTAD), Quinta de Prados, 5000-801 Vila Real, Portugal; avalente@utad.pt; 2 ENEIDA, Grid Intelligence, S.A., Rua Alexandre Herculano 21B, 3000-019 Coimbra, Portugal; joliveira@eneida.io; 3 Coimbra Institute of Engineering, Polytechnic University of Coimbra, Rua Pedro Nunes-Quinta da Nora, 3030-199 Coimbra, Portugal; vsantos@isec.pt; 4 INESC Coimbra, DEEC, Polo II, 3030-290 Coimbra, Portugal; 5 INESC Technology and Science, 4200-465 Porto, Portugal

**Keywords:** runtime firmware update, smart sensors, Internet of Things, real-time systems, concurrent tasks, in-application programming

## Abstract

Smart sensor devices increasingly require remote firmware updates to deploy new functionality, security patches and algorithmic improvements without interrupting services. Runtime firmware updates remain a significant challenge in embedded sensing systems, particularly in real-time and high-availability applications where service interruption and system reboot are undesirable. This paper presents an innovative runtime firmware update mechanism for 32-bit embedded systems architecture and integrated with a custom Runtime Scheduler (RTS). The proposed approach enables dynamic Flash Program Memory (FPM) reprogramming without reboot while preserving concurrent task execution, making it suitable for intelligent sensors and edge-based IoT devices requiring continuous operation. Three update granularities, application, function and row levels, were implemented and experimentally evaluated. Reducing update granularity significantly decreased both system downtime and update data size. For the demonstration update developed to validate the concept, function-level updates reduced the transmitted patch size to approximately 35% of the data that would have been required for the equivalent application-level update, while row-level updates reduced the maximum continuous system unavailability time to approximately 16 ms by distributing FPM write operations across multiple RTS cycles. The mechanism also incorporates instruction cache invalidation, dynamic task validation and fault recovery procedures. Experiments using an intelligent power quality monitoring sensor testbed demonstrate its feasibility while improving availability, reducing update overhead and maintaining operational continuity.

## 1. Introduction

Embedded systems are currently widely integrated into smart sensors, Internet of Things (IoT) infrastructures, cyber-physical systems, industrial automation, and real-time monitoring platforms, where uninterrupted operation and remote maintenance play a critical role. In these environments, firmware updates are essential not only for correcting vulnerabilities and software defects, but also for enabling the introduction of new functionalities and enhancing the system’s ability to adapt its behaviour dynamically, thereby extending the operational lifetime of devices after deployment [1,2,3,4].

Modern smart sensors increasingly rely on 32-bit microcontrollers to implement advanced sensing functionalities that exceed the capabilities of traditional low-complexity platforms. The increased computational performance, larger memory resources, and integrated communication peripherals available in contemporary 32-bit devices have enabled their adoption in intelligent power quality analysers, smart energy meters, industrial condition monitoring systems, environmental monitoring nodes, biomedical instrumentation, and distributed IoT sensing platforms. These applications frequently combine data acquisition, digital signal processing, communication services, and local decision-making within a single embedded device [5].

These sensing platforms have also become increasingly complex in terms of software architecture, requiring the execution of multiple concurrent tasks responsible for data acquisition, signal processing, data logging, communications, and system diagnostics [5]. As many of these sensors are expected to operate continuously and are frequently deployed in remote or difficult-to-access locations, firmware maintenance, security patching, and functional upgrades should ideally be performed without interrupting normal operation or compromising service availability [3,4,6,7].

Traditional firmware update mechanisms are predominantly based on bootloaders responsible for receiving, validating, and installing new firmware images, with complete replacement of the existing firmware image being the most common update strategy [3,4,8,9,10]. Despite their robustness and relative ease of implementation, these approaches typically require application interruption and system reboot to activate the new firmware version [6,11,12]. This requirement results in periods of system unavailability and loss of the execution context, representing a significant limitation in smart sensors, IoT devices, and other embedded systems with high-availability requirements or stringent timing constraints.

For sensing applications that require continuous data acquisition or uninterrupted monitoring services, even short periods of downtime may lead to data loss, reduced measurement reliability, or temporary loss of system observability.

To mitigate these impacts, several studies have explored techniques such as incremental updates, differential firmware transmission (delta updates), code compression, modular updates, and hotpatching mechanisms [7,8,13,14,15,16,17,18,19]. In parallel, recent research has begun to investigate runtime update approaches capable of partially modifying firmware without requiring explicit system reboot [5,9,10,20]. However, most of these approaches remain limited to systems with low execution complexity, often assuming exclusive control of the microcontroller, the absence of significant concurrency, or the imposition of temporary restrictions on the normal execution of the application during the update process. These assumptions are increasingly incompatible with modern smart sensors and edge IoT devices, which commonly execute concurrent sensing, processing, and communication tasks.

Despite the progress achieved, significant gaps remain in the literature regarding the application of runtime updates in modern 32-bit microcontrollers executing multitasking systems. Existing studies rarely address issues explicitly related to cache coherence, synchronisation of concurrent tasks, atomicity of code replacement operations, and preservation of system integrity during Flash Program Memory (FPM) reprogramming procedures [6,13,21]. These limitations become particularly critical in multitasking embedded architectures, including smart sensors and edge IoT devices, where multiple tasks may simultaneously access the code and data regions affected by the update process.

In this context, this paper proposes a partial runtime firmware update mechanism for multitasking embedded sensing systems implemented on the PIC32MZ (Microchip Technology Inc., Chandler, AZ, USA) microcontroller family [22] and integrated with a proprietary Runtime Scheduler (RTS). The proposed mechanism enables dynamic reprogramming of the FPM without requiring system reboot, while preserving the concurrent execution of the remaining system tasks.

To evaluate the impact of different firmware update granularities, approaches ranging from complete application replacement to the selective update of individual components stored in the FPM were implemented and experimentally evaluated. The proposed mechanism further introduces a functional memory organisation strategy based on linker script-controlled segmentation, explicit cache coherence mechanisms, dynamic validation of update integrity, and an automatic fault recovery mechanism for runtime reprogramming operations.

The remainder of this paper is organised as follows. Section 2 presents the state of the art and discusses the main firmware update mechanisms for embedded systems. Section 3 describes the architecture of the proposed mechanism and the implemented partial update strategies. Section 4 presents the experimental results obtained. Section 5 discusses the results and compares the developed mechanism with the state of the art. Finally, Section 6 presents the main conclusions and outlines directions for future work.

## 2. Related Work

Firmware updates constitute an essential mechanism for ensuring the security, maintenance, and functional evolution of embedded systems and IoT devices. In traditional approaches, new firmware versions are distributed through Over-The-Air (OTA) mechanisms and installed by dedicated bootloaders, typically relying on complete replacement of the firmware image. Following FPM programming, these conventional approaches usually require a system reboot to activate the new firmware version [3,4,7]. In contrast, few studies address firmware modification during runtime without requiring a system reboot [1,2,6,12,13]. Although widely adopted, they introduce periods of system unavailability and entail the loss of the application’s execution state, representing a significant limitation in real-time systems and safety-critical applications.

In recent years, research has focused on reducing the volume of transmitted data, energy consumption, and system downtime. To this end, incremental update, differential compression, and hot patching techniques have been proposed [8,14,16,17,18]. More recently, runtime update mechanisms have emerged that are capable of partially modifying firmware without requiring an explicit system reboot [1,2,6,12,13]. However, many of these solutions remain limited to systems with low execution complexity and do not explicitly address issues related to cache coherence, synchronisation of concurrent tasks, and atomicity in Real Time Operating System (RTOS) based multitasking systems.

The studies by Bauwens et al. [3], Arakadakis et al. [4], and El Jaouhari and Bouvet [7] indicate that the majority of OTA mechanisms still depend on full firmware replacement followed by a system reboot. Although these approaches have introduced improvements in security, interoperability, and energy efficiency, they do not consider continuous runtime execution mechanisms or explicitly address the challenges related to cache coherence and task concurrency in 32-bit systems.

Several authors have explored differential update techniques to reduce the volume of transmitted data and the number of write operations required during firmware updates. Bogdan et al. [16], Ji et al. [10], and De Simone et al. [8] employ delta patching mechanisms that transmit only the binary differences between successive firmware versions. Similarly, Wee and Kim [17] use compression techniques to reduce the size of Firmware Over-The-Air (FOTA) updates. In parallel, Wei et al. [14] address the challenge of updating batteryless devices by proposing an energy-efficient mechanism based on incremental updates.

Although these approaches significantly improve energy efficiency and reduce net-work traffic, they remain dependent on bootloaders and ultimately require a system reboot to complete the update process. Furthermore, these studies do not address the synchronisation and cache coherence mechanisms required by modern 32-bit architectures.

Other studies have investigated approaches based on modularisation and partial code replacement. Kwon et al. [20] propose an update strategy based on function blocks and pointer redirection, enabling only modified components to be updated and thereby reducing the memory and energy overhead associated with the update process. Similarly, Xia et al. [5] present an architecture based on operating modes that supports the dynamic replacement of software modules in wireless sensor network nodes, thereby enabling functional reconfiguration of the system without requiring the complete reinstallation of the firmware.

Although these approaches contribute to reducing the amount of updated code and the costs associated with the reprogramming process, they focus primarily on software modularisation and the partial replacement of components. As a result, issues such as cache coherence, synchronisation among concurrent tasks, and the implementation of persistent runtime updates to program memory are not adequately addressed, particularly in RTOS-based multitasking systems and 32-bit architectures.

The studies most closely related to this research focus on hot patching and live updating mechanisms, which are techniques that enable parts of an application’s code to be modified or replaced during execution without requiring system interruption or a complete reboot. Neves et al. [1,2] demonstrate the feasibility of partial runtime code replacement using absolute addressing and direct reprogramming of program memory in 8-bit PIC18 microcontrollers [23]. However, the authors explicitly assume the absence of compatibility with RTOSs and do not provide support for systems featuring hardware abstraction layers or concurrent tasks.

Uven et al. [6] propose “Append Update”, an approach based on the atomic replacement of branch instructions in 32-bit systems. Zhou et al. [12] introduce StackPatch for ARM, RISC-V, and Xtensa architectures, enabling dynamic redirection of execution flow through stack frame reconstruction. Kim et al. [13] present Kintsugi, a secure hotpatching framework for real-time systems based on code shadowing.

The state-of-the-art analysis indicates that most existing solutions still require a system reboot after the update process, even when differential or modular mechanisms are employed to reduce the volume of transmitted data and update time. More recent studies on hotpatching and live updating have explored partial runtime code replacement; however, they remain limited in terms of support for 32-bit microcontrollers with cache memory, automatic synchronisation of concurrent tasks, and native integration with RTOSs.

Significant gaps therefore remain in ensuring cache coherence following program memory reprogramming, guaranteeing atomicity during code replacement, and preserving the temporal integrity of the system during concurrent updates. Furthermore, many existing mechanisms assume exclusive control of the processor, rely on volatile RAM, or require explicit programmer intervention to maintain the functional correctness of the system throughout and after the update process.

In this context, this work advances the state of the art by proposing a partial firmware update mechanism. The proposed mechanism incorporates explicit cache coherence management, synchronisation of concurrent tasks in RTOS based systems, dynamic Flash Program Memory reprogramming, and automatic failure recovery, thereby ensuring operational continuity and preserving system integrity throughout the update process.

## 3. Proposed Mechanism

The proposed mechanism extends previous runtime firmware update approaches developed for low-complexity microcontrollers [1,2] to a 32-bit multitasking platform based on the PIC32MZ2048EFM100 (Microchip Technology Inc., Chandler, AZ, USA) microcontroller [22]. Unlike previous approaches, this work addresses partial firmware updates during the concurrent tasks execution in instruction cache based architectures. This requires the use of explicit cache management mechanisms and task validation at the scheduler level to maintain execution consistency during FPM reprogramming. The transition from 8-bit architectures to a modern 32-bit platform introduces additional challenges related to cache coherence, execution synchronisation, and temporal predictability.

To support selective firmware replacement, the memory layout is based on the static and absolute allocation of functions within the FPM. This approach prevents the automatic relocation of functions across different firmware versions and ensures deterministic placement of executable code in program memory. As a result, only the modified memory regions need to be reprogrammed during the update process. In the PIC32MZ [22], the FPM is organised into pages and programming rows, which correspond to the minimum erase and write units, respectively, supported by the Non-Volatile Memory (NVM) controller. Table 1 summarises the Flash Program Memory organisation of the PIC32MZ microcontroller [22] employed in this study.

Since erase operations affect entire program memory pages, minor firmware modifications may temporarily invalidate memory regions that are considerably larger than the data being updated. To mitigate this limitation, the proposed mechanism combines modular FPM segmentation with partial reprogramming strategies executed progressively during runtime.

The PIC32MZ2048EFM100 [22] was selected as the experimental platform because it is a 32-bit microcontroller architecture featuring an instruction cache and in-application programming capability. This enables firmware to be modified without requiring the device to exit its normal execution mode. The platform therefore provides an ideal environment for evaluating the additional challenges introduced by instruction cache management during runtime Flash memory reprogramming in a 32-bit architecture with concurrent task execution.

The selected microcontroller [22] has a 16 KB instruction cache and a 4 KB data cache. Its program flash memory is organised into 16 KB pages and 2 KB programming rows. This organisation is directly relevant to the proposed mechanism, as page erasure and row programming define the physical granularity of flash operations, while the proposed approach additionally introduces a logical segmentation of the rows into 128-byte regions, allowing the programming operations to be distributed across multiple RTS cycles.

This microcontroller [22] is not representative of all 32-bit microcontrollers with cache. Instead, it is used as a specific platform on which the proposed mechanism and its associated cache management and runtime execution requirements can be evaluated experimentally. It can be adapted to other architectures where equivalent In-Application Programming mechanisms for Flash memory are available. However, implementation requires adaptations to the target device’s memory organisation, flash programming and erase granularity, Read-While-Write capabilities, and the cache architecture.

To support the selective transmission of only the modified program memory blocks, an adapted version of the Intel HEX format was employed, incorporating additional in-formation regarding target region addressing, update process delimitation, and integrity verification. The integrity of the received data is verified at two levels. Each line of the patch HEX file is accompanied by a checksum, allowing block-level integrity errors to be detected during reception. Additionally, a CRC is calculated for the entire patch file, allowing the overall integrity of the received data to be validated before the update is applied. This mechanism is intended to detect corruption or accidental alteration of the data, it does not constitute source authentication mechanism for the firmware.

The patch generation procedure and the Intel HEX format extensions used in the proposed implementation are based on the mechanisms outlined in references [1,2] and have been modified for the use with PIC32MZ2048EFM100 [22] platform.

Dynamic FPM updates in 32-bit architectures introduce additional challenges associated with cache coherence and concurrent task execution. Unlike architectures without cache mechanisms, the PIC32MZ [22] incorporates an Instruction Cache (I-cache) and a Data Cache (D-cache) between the CPU and program memory. Following FPM reprogramming operations, instructions previously stored in the cache may remain invalid, potentially leading to the execution of outdated code.

The tests showed that the absence of explicit I-cache invalidation may result in incorrect execution after FPM reprogramming. This behaviour is associated with the persistence of obsolete instructions that are stored in the I-cache persistently, even after the corresponding program memory regions have been modified. Following the introduction of the proposed I-cache invalidation and synchronisation procedure, the updated firmware was consistently executed correctly. These observations support the inclusion of explicit I-cache management mechanisms in the proposed approach.

To prevent this issue, the proposed mechanism explicitly performs I-cache synchronisation and invalidation operations after each FPM write operation. The procedure temporarily disables the prefetch mechanism, invalidates the cache rows associated with the modified region, and executes synchronisation barriers before returning to normal execution. In addition, critical non-volatile memory handling routines are executed from non-cacheable regions (kseg1), thereby reducing temporal inconsistencies during runtime updates.

The D-cache is not explicitly invalidated during the firmware update, as this FPM update operation modifies executable code rather than data stored in the processor cache. D-cache maintenance is relevant when the data structures involved in the update are shared with other memory access agents, such as DMA, or when coherence of the D-cache with respect to those data must be explicitly ensured.

The firmware update mechanism was integrated into a proprietary Runtime Scheduler, designed to provide a controlled and deterministic execution environment. In addition to task scheduling, the RTS monitors task deadlines and their respective temporal constraints. It can also suspend non-essential tasks that exceed their deadlines in order to prioritise essential tasks. During the update process, the RTS verifies each task’s callback address and the logical state of the associated FPM region. This prevents the execution of tasks whose code is located in an erased or partially programmed region. Update operations can be distributed across multiple RTS execution cycles, enabling unaffected tasks to continue executing.

Using a proprietary RTS in the developed solution does not imply compatibility with conventional general-purpose RTOSs, since the proposed mechanism has not been validated on platforms such as FreeRTOS [24] or Zephyr [25].

The Runtime Scheduler employs a dynamic scheduling policy based on the nearest deadline. Eligible tasks are ordered according to their respective deadlines. Each task has an individual countdown timer that determines the instant it becomes eligible for execution, with scheduling decisions being made periodically within time slots defined by the system. As task execution is non-preemptive, the execution time consumed by each task during each scheduling cycle can be determined.

When a task fails to meet its deadline, the RTS signals the occurrence and evaluates the task’s criticality. Non-essential tasks that exceed their deadlines are removed from the scheduler, preventing them from compromising the execution of essential tasks. Essential tasks remain under the management of the RTS and continue to be considered during the scheduling process. The firmware update process is treated as an essential task, ensuring that the operations required for the update remain active throughout the update process.

**Figure 1:**
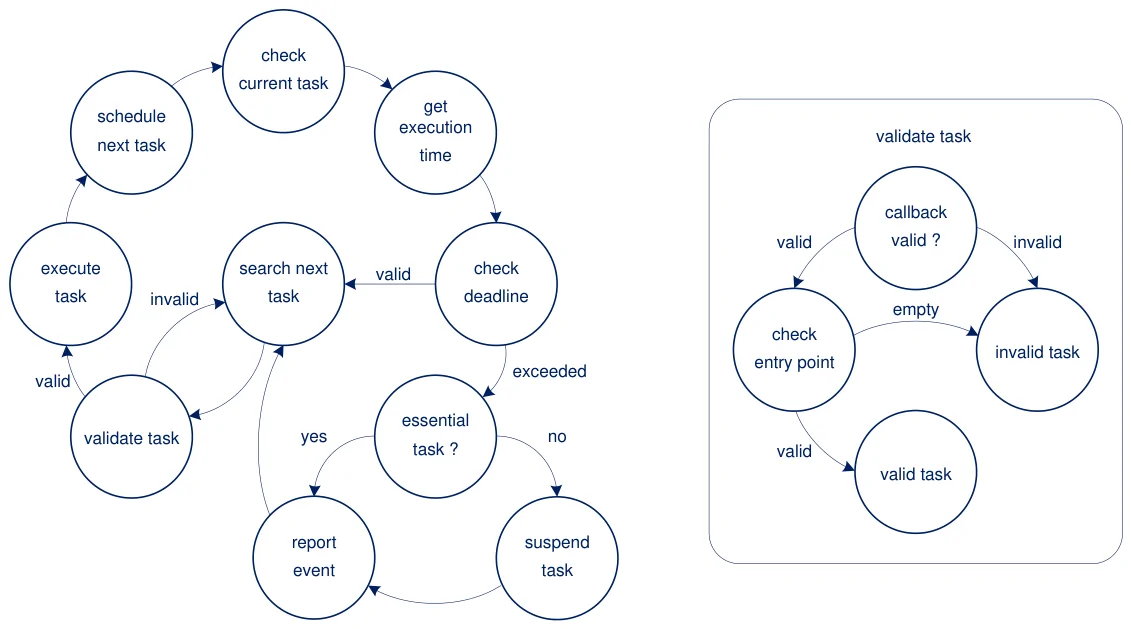
Implemented runtime scheduler and validation mechanism.

During the update process, only the FPM regions undergoing modification become temporarily unavailable, while the remaining applications continue operating normally. To prevent the execution of invalid code, the RTS validates not only the callback address of each task but also the logical state of the corresponding FPM region before activating it. If the memory region is found to be erased or partially programmed, the associated task is temporarily excluded from scheduling until the update process has been completed. Furthermore, as illustrated in Figure 1, the RTS monitors task execution timing and verifies the validity of the associated memory region before considering tasks eligible for execution, thereby ensuring that only functionally consistent tasks satisfying their temporal constraints remain active during the reprogramming process.

The same validation principle applies to interrupt service routines, callbacks, and other asynchronous events. The existence of an interrupt vector or a pointer to a service routine does not automatically result in its execution. Before making the routine available for execution, the system verifies the address of its respective entry point and the validity of the associated code. If the routine is located in an erased, partially programmed, or temporarily unavailable FPM region, its execution is prevented until the region has been completely reprogrammed and validated. This ensures that the occurrence of asynchronous events during an update does not lead to the execution of inconsistent code, maintaining the protection mechanism regardless of the peripheral or application that generates the event.

**Figure 2:**
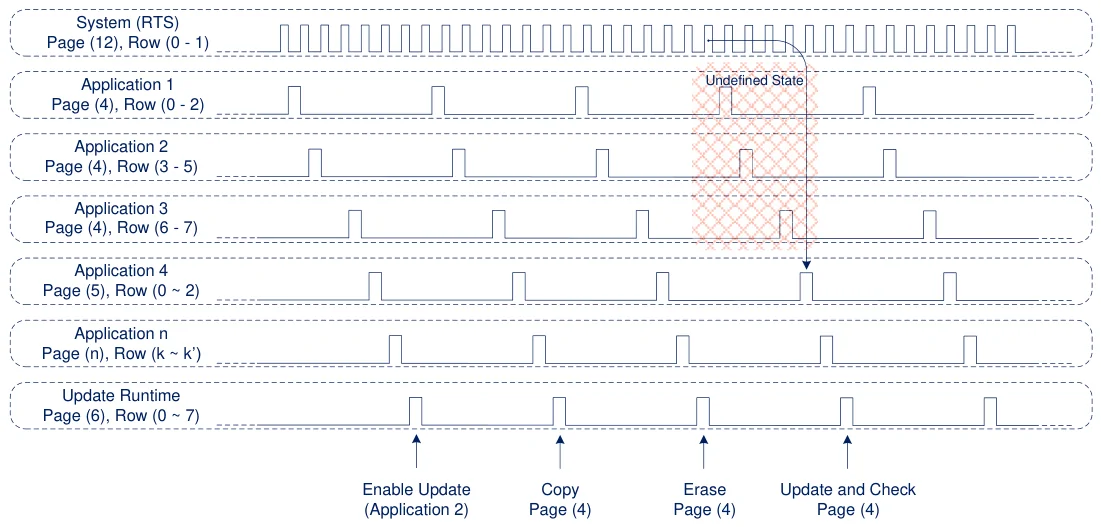
Timing diagram of the RTS managed task scheduling during firmware update process and FPM reprogramming.

Figure 2 illustrates the interaction between the proposed firmware update application and the task scheduling managed by the RTS. The update process is distributed across multiple execution cycles, ensuring that only the FPM page undergoing reprogramming becomes temporarily unavailable. During this interval, indicated by the shaded region, tasks whose functions reside in the affected page are considered ineligible for execution, whereas the remaining applications continue to be scheduled normally. This approach confines the impact of the update process to the region being modified, thereby preserving overall system availability and avoiding disruption to the remaining system functionalities during reprogramming.

This approach enables partial runtime firmware updates while preserving the stability of the scheduler and the concurrent execution of unaffected tasks. This ensures the system’s overall operational continuity.

The proposed method performs selective FPM reprogramming during normal system operation using the internal NVM controller. Instead of replacing the entire firmware image, only the modified regions of program memory are updated, thereby reducing both system downtime and the volume of transmitted data.

**Figure 3:**
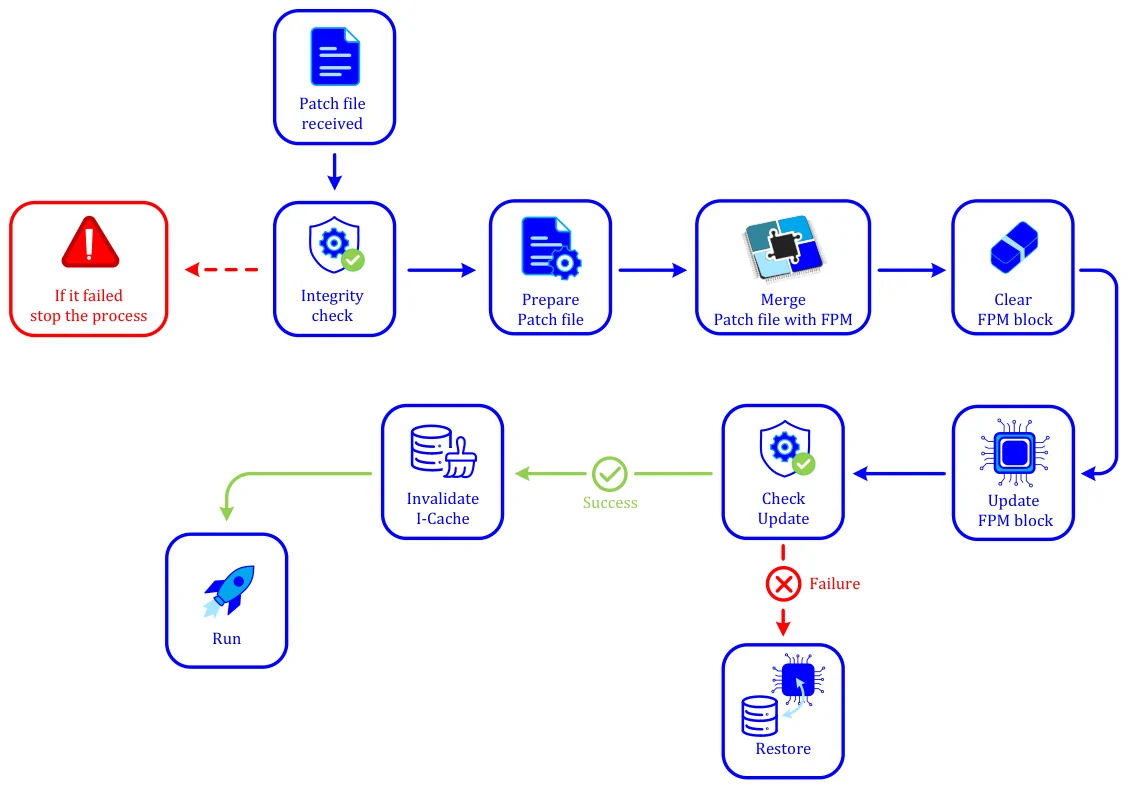
Flowchart of the proposed runtime firmware update algorithm.

Figure 3 illustrates the operational flow of the proposed runtime partial firmware update mechanism for the PIC32MZ architecture. The process begins with the reception and integrity verification of the firmware patch to be updated. This is followed by its preparation and integration with the affected FPM regions. The selected FPM blocks are subsequently erased and progressively reprogrammed, with the result of each stage being validated before proceeding. In the event of a failure, the recovery procedure is triggered to restore a valid state. After successful reprogramming, the instruction cache is explicitly invalidated before the updated code is made available for execution. The flow presents the operational sequence of the update, and the validation, recovery, and cache management mechanisms required to preserve execution consistency during runtime reprogramming.

Once the patch file has been received and its integrity verified, the “Prepare Patch File” stage shown in Figure 3 organises the received content according to the program memory organisation. The data are structured into rows corresponding to the FPM regions to be updated. Two row structures are created in RAM during this stage: one containing the current FPM content and used as the backup copy, and another containing the content corresponding to the received patch. The following stage merges these two structures according to the FPM organisation. This preserves the original content in the positions not covered by the patch, while applying the content from the most recent firmware version to the modified positions. The resulting structure represents the final content of the rows that will subsequently be used for FPM reprogramming.

A copy of the original FPM content is stored in the backup structure in RAM throughout the update process. This copy constitutes the reference state for recovery in the event of a failure during reprogramming or during the final validation. If a failure is detected, the content of the backup copy is restored to the corresponding FPM region and subsequently verified.

Three distinct strategies for partial runtime FPM updates were implemented and experimentally evaluated:

(i) App-level updates;
(ii) Function-level updates;
(iii) Row-level updates.

These strategies differ primarily in the amount of memory reprogrammed during each RTS iteration and in the associated trade-offs between temporal impact, implementation complexity, and application availability.

The app-level strategy performs the complete reprogramming of an FPM page during a single RTS iteration. After copying the original contents, the corresponding page is erased and fully reprogrammed before normal execution resumes.

This approach offers the simplest implementation and reduces the likelihood of applications remaining in a partially programmed state. However, because all write operations are executed consecutively within the same scheduling cycle, this strategy introduces the highest execution-time overhead per iteration and temporarily increases system latency.

The function-level strategy corresponds to the second approach analysed and is based on the functional segmentation of sub-applications through the linker script. This approach enables each function to be positioned in specific and deterministic regions of the FPM, thereby preserving structural stability across different firmware versions.

Although the physical erase and reprogramming operations continue to be performed at the page level, the update process targets only the functional blocks that have effectively been modified. As a result, the amount of code transmitted and processed during the update process is reduced.

**Figure 4:**
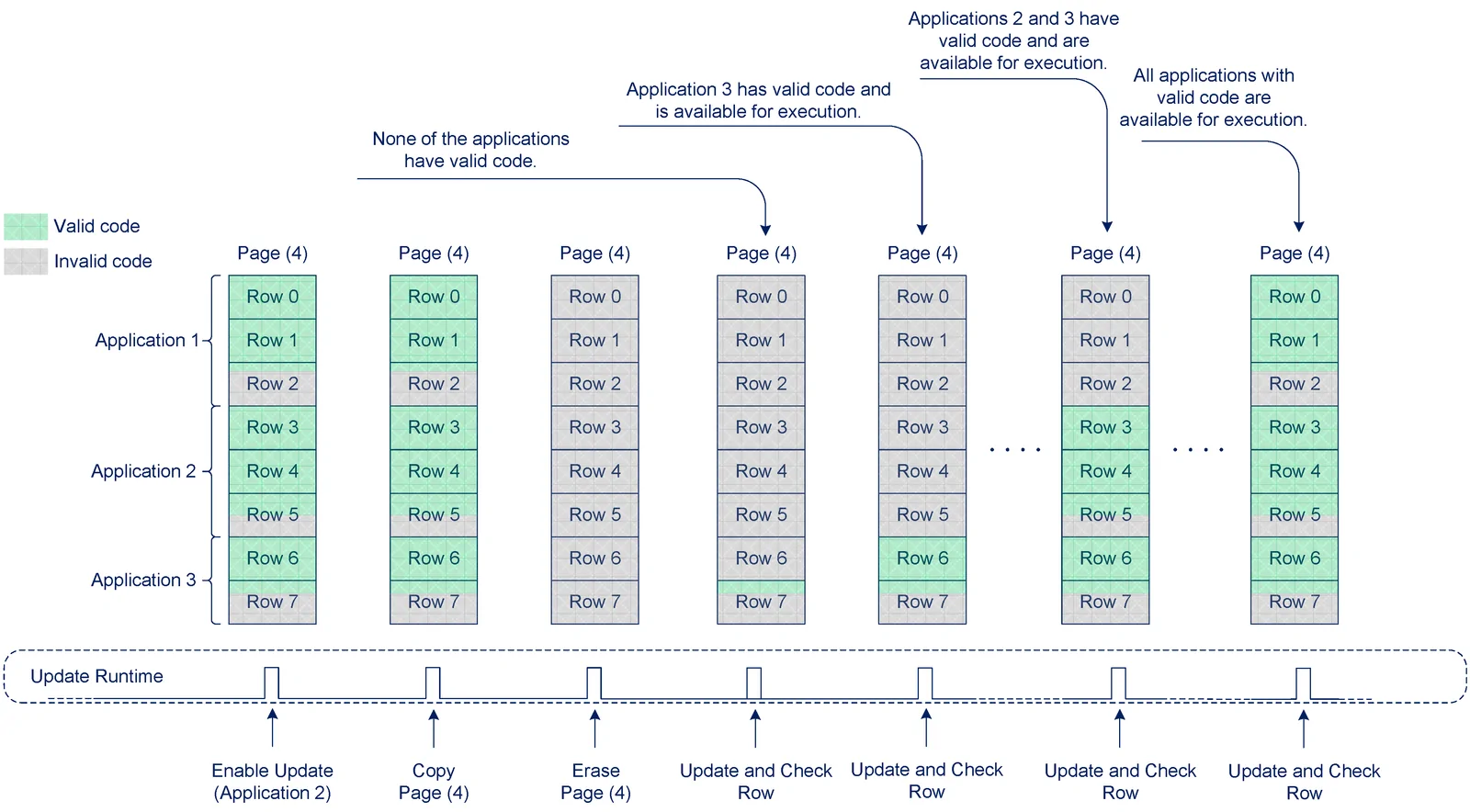
Proposed phased update process of an FPM page.

To prevent the execution of partially programmed applications, the FPM rows are reprogrammed in reverse order, ensuring that the application entry point becomes valid only after the corresponding functional block has been fully updated. As illustrated in Figure 4, the code regions are updated from the highest-addressed rows to the lowest addressed rows, with Row 0 being programmed only during the final stage of the process. Consequently, the application remains unavailable for execution while any portion of its code is incomplete, becoming eligible for scheduling only after the update and validation of the corresponding functional block have been successfully completed.

The row-level strategy represents the finest-grained FPM reprogramming approach implemented in the system. In this strategy, only a single FPM row is programmed and validated during each RTS iteration. Distributing the reprogramming process across multiple consecutive cycles significantly reduces the temporal impact introduced in each iteration and minimises interference with the remaining concurrent tasks. However, because applications remain partially unavailable for a longer period following page erasure, this approach introduces the largest temporal window of program memory inconsistency. Table 2 summarises the main characteristics of the implemented partial update strategies.

During the development of the proposed method, a limitation was identified when the code associated with a sub-application to be updated spans multiple FPM pages. As illustrated in Table 3, the sub-application *Sensor_Node* partially occupies pages 4 and 5 of program memory. This situation poses a significant risk during the update process, as application-level updates are performed at page granularity.

**Figure 5:**
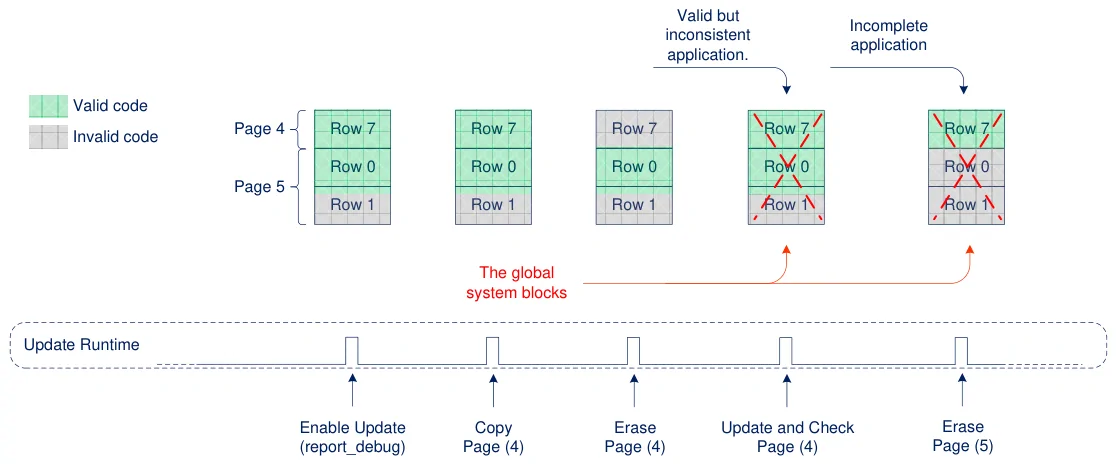
Phased update process problems that occur when a sub-application spans multiple FPM pages.

In this scenario, both the end of page 4 and the beginning of page 5 would need to be updated simultaneously. However, because the RTS executes tasks in real time, updating only one of these pages would temporarily leave the sub-application in an inconsistent state. Such inconsistency could result in the execution of incomplete code, unpredictable behaviour of the associated task, or even compromise overall system stability (Figure 5). Consequently, the RTS would have no means of guaranteeing system integrity until the second page had also been fully updated.

To overcome this limitation, the memory layout was designed to ensure that each sub-application remains entirely contained within a single FPM page. In cases where a sub-application exceeds the size of a single page, its code must be structured modularly to ensure that updating one page does not compromise the code residing in another. To this end, critical functions and entry points should be isolated within a single page, thereby minimising dependencies spanning pages updated at different times.

Additionally, functions invoked outside the task callback are validated through the address of their respective entry point and the associated code content before execution, preventing calls to functions located in erased or partially programmed FPM regions.

The combination of modular FPM segmentation, static function allocation, explicit cache coherence mechanisms, and validation integrated within the RTS enables runtime firmware updates in 32-bit multitasking embedded systems without requiring system reboot or complete firmware replacement.

## 4. Results

**Figure 6:**
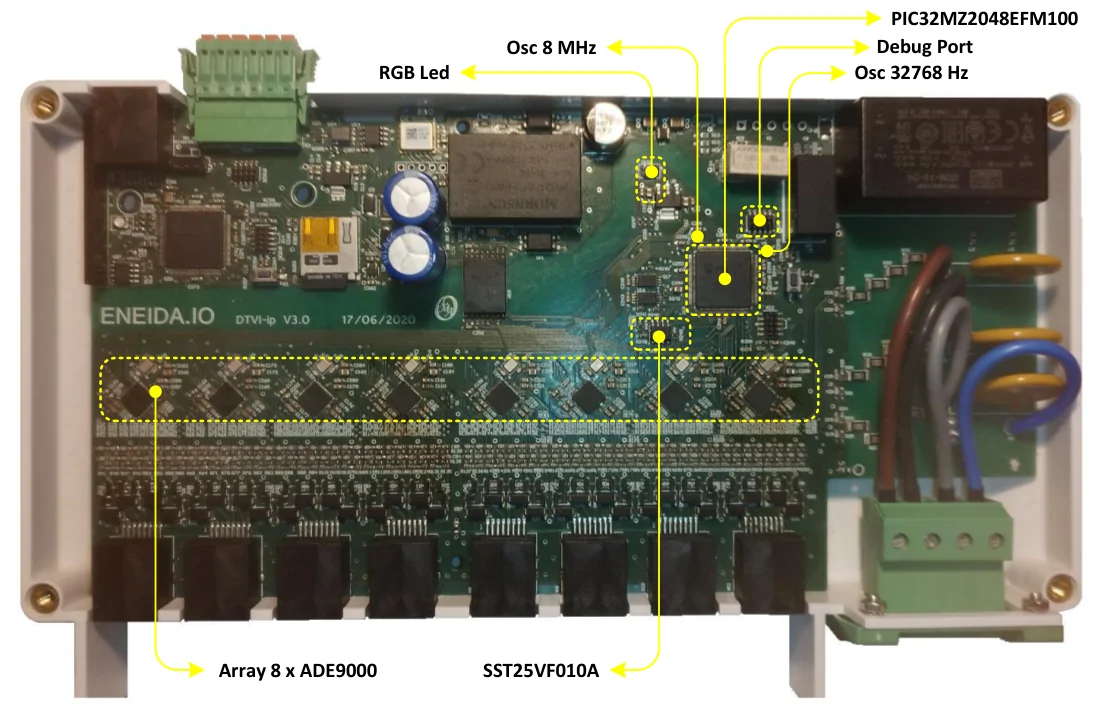
DTVI smart sensor used as a testbed to validate the proposed runtime firmware update mechanism.

To experimentally validate the proposed runtime firmware update mechanism, the DTVI smart sensor, developed by Eneida Grid Intelligence S.A. [26], was employed. Designed for low-voltage electrical network monitoring applications, this device integrates a diverse set of peripherals and critical functionalities whose continuous operation is essential to overall system reliability. In this context, the need to preserve operational availability throughout the update process makes this equipment a particularly relevant case study for evaluating the proposed approach. Figure 6 presents the DTVI smart sensor used in the experimental validation of the proposed mechanism.

The central element of the architecture is the PIC32MZ2048EFM100 [22] microcontroller from Microchip Technology Inc., based on the MIPS32 architecture. This device operates at frequencies of up to 200 MHz and integrates 2 MB of internal Flash memory for code storage and 512 KB of RAM. It provides a wide range of communication interfaces, timers, analogue-to-digital converters (ADrCs), and a real-time clock module, offering resources well suited to embedded applications with parallel processing and stringent timing requirements. The acquisition and conditioning of the electrical signals are performed by eight ADE9000 [27] analogue front-end devices, connected to the microcontroller via SPI.

To assess the correct operation of the board during end-of-line testing, an application designated as app_production_test was developed. This application comprises several sub-applications, including one for verifying and validating the status of the external oscillators, another for validating the operational status of the analogue front-end devices, and a third for validating the external Flash memory.

The hardware platform executes multiple concurrent sub-applications under the control of the RTS, including hardware monitoring, analogue signal acquisition, oscillator supervision, diagnostic, and event communication modules.

To validate the update mechanism, a functional modification was introduced into the hardware_monitor sub-application, which is responsible for the visual indication of the system status. In the original firmware version, correct system operation was indicated by a green LED, whereas in the modified version the indication was provided by a blue LED. This modification enabled the correct application of the updates to be visually verified during normal system operation.

The three update strategies previously presented were then evaluated experimentally. The analysis focused on the temporal behaviour of the RTS, the impact of the different strategies on system downtime and the volume of transferred data, the availability and functional coherence of the applications during FPM reprogramming, and the robustness of the mechanism under failure conditions.

The first strategy analysed corresponded to the application-level update strategy, in which an entire FPM page is reprogrammed during the update process. Table 4 presents the program memory organisation employed in the experimental trials, showing that multiple sub-applications simultaneously shared the same FPM page.

The comparison between the original and modified firmware versions showed that the changes introduced within a function of the hardware_monitor sub-application were confined to the memory region associated with that sub-application, and no changes were observed in the remaining program memory regions. This behaviour makes it possible to identify the FPM region affected by the update in a deterministic manner.

**Figure 7:**
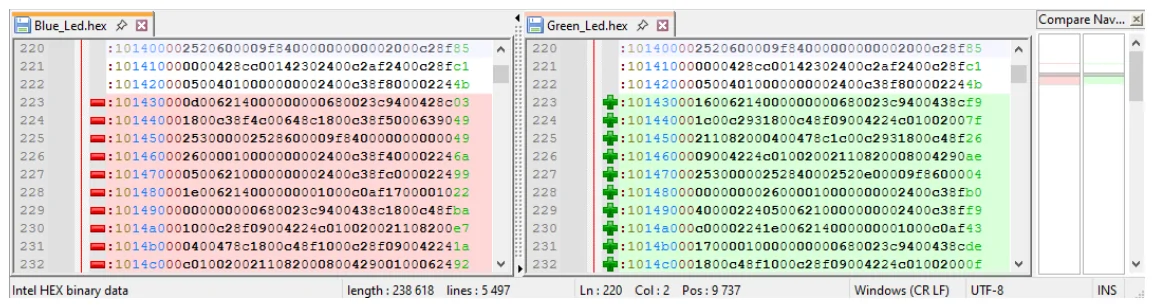
Modified program memory area during the firmware update process, visualised from the Intel Hex File.

Figure 7 presents the comparison between the Intel HEX files corresponding to the two firmware versions, highlighting the lines modified between the two versions. Based on the FPM organisation presented in Table 1 and the memory allocation presented in Table 4, the hardware_monitor sub-application is located in the region between 0x0011000 and 0x0012000, obtained from the combination of the page identifier, the page size, and the row identifier. The changes observed in Figure 7 are therefore associated with the memory region allocated to the hardware_monitor sub-application, while the remaining regions remain unchanged.

This result highlights the main characteristic of the update strategy at the application level: although the functional change is located in a specific part of the firmware, the update unit remains constrained by the organisation of the program memory, making it necessary to consider the FPM region corresponding to the affected application.

The generated update file included only the records corresponding to the target FPM page, thereby reducing the volume of transmitted data compared with complete firmware replacement. Following validation of the update file, the system successfully executed the entire page reprogramming process without requiring a firmware reboot.

**Figure 8:**
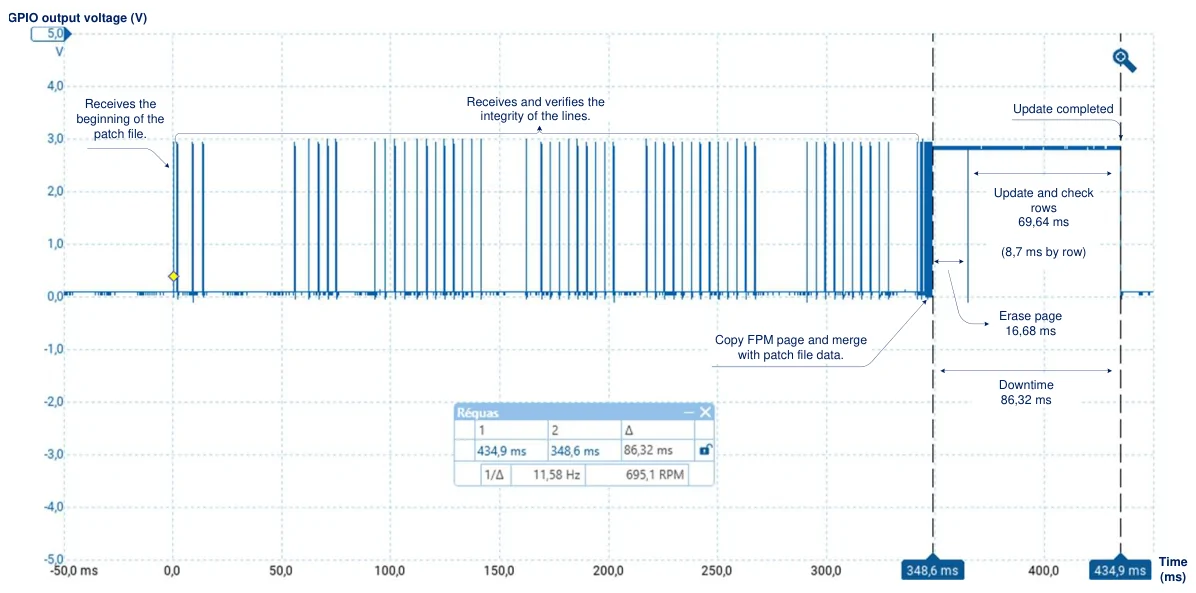
Timing diagram illustrating the different stages of the firmware update process using the application-level update strategy.

Figure 8 illustrates the timing diagram associated with the firmware update process. A signal was asserted whenever the update application took control of the microcontroller, thereby indicating the instant at which the Intel HEX update file was received, as well as the periods during which the erase, programming, and validation operations were performed. Across all experimental trials, an average page erase time of approximately 16.68 ms and an average row programming time of 8.7 ms were observed, resulting in a maximum continuous period of unavailability of approximately 86.32 ms. During this interval, the FPM page undergoing the update remained temporarily unavailable for execution.

The second strategy analysed corresponds to the function-level update strategy employing app-level programming. In this approach, the program memory organisation was restructured based on the functional segmentation of sub-applications using the linker script, enabling each function to be allocated to specific and deterministic regions of the FPM. To experimentally evaluate this strategy, modifications were introduced into the hardware_monitor_check function of the hardware_monitor sub-application. Table 5 presents the program memory footprint associated with selected functions of the hardware_monitor sub-application.

The comparison between the original and modified firmware versions confirmed that the changes were confined exclusively to the memory region corresponding to the modified function, with no modifications observed in the remaining adjacent functions within the same sub-application. The Intel HEX files, memory map files, and linker scripts used in this study, can be found in more detail in the Supplementary Materials.

**Figure 9:**
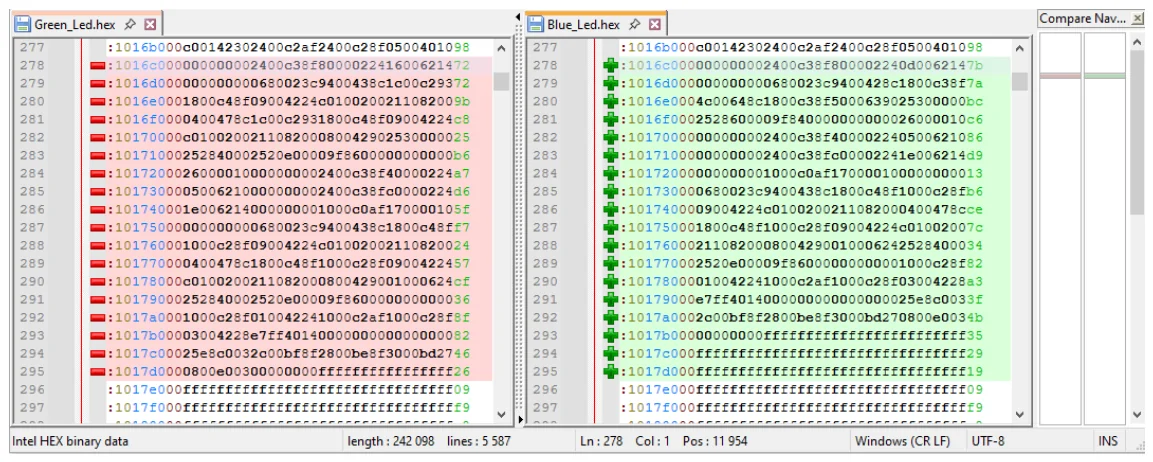
Program memory region corresponding to the hardware_monitor_check function after functional segmentation, visualised from the corresponding Intel HEX file.

Figure 9 illustrates the hexadecimal region associated with the hardware_monitor_check function after the application of functional segmentation.

**Figure 10:**
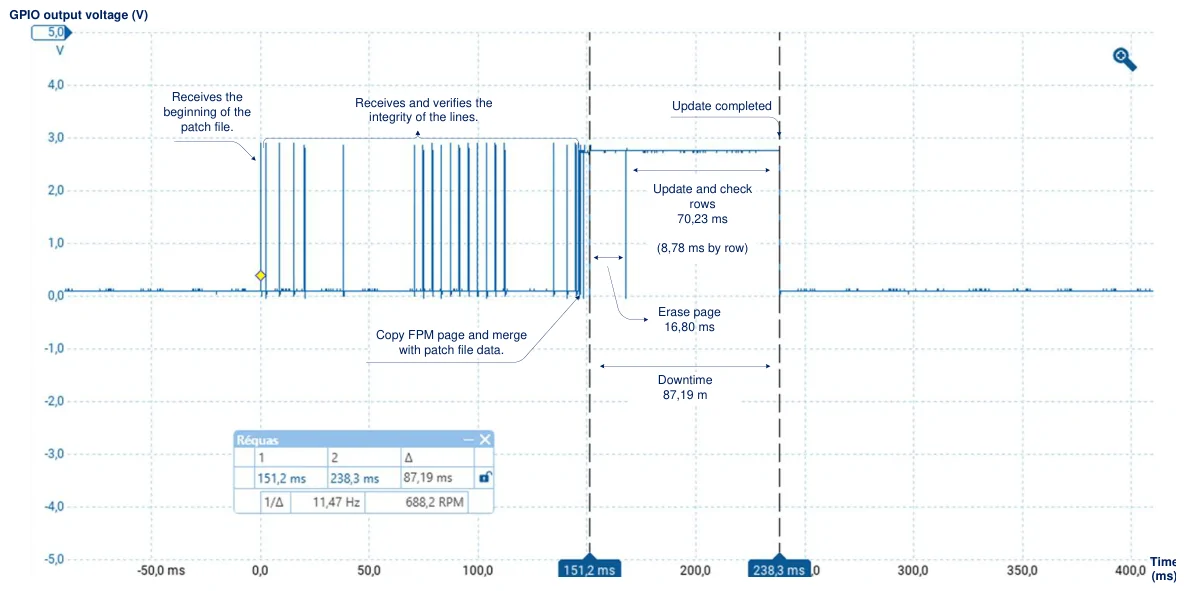
Timing diagram illustrating the firmware update process for the function-level update strategy employing app-level programming.

Compared with the previous strategy, the timing associated with FPM erase and programming operations remained largely unchanged. Experimentally, an average page erase time of approximately 16.80 ms and an average row programming time of 8.78 ms were observed, resulting in a maximum continuous period of unavailability of approximately 87.19 ms. The temporal behaviour of this strategy is illustrated in Figure 10.

**Figure 11:**
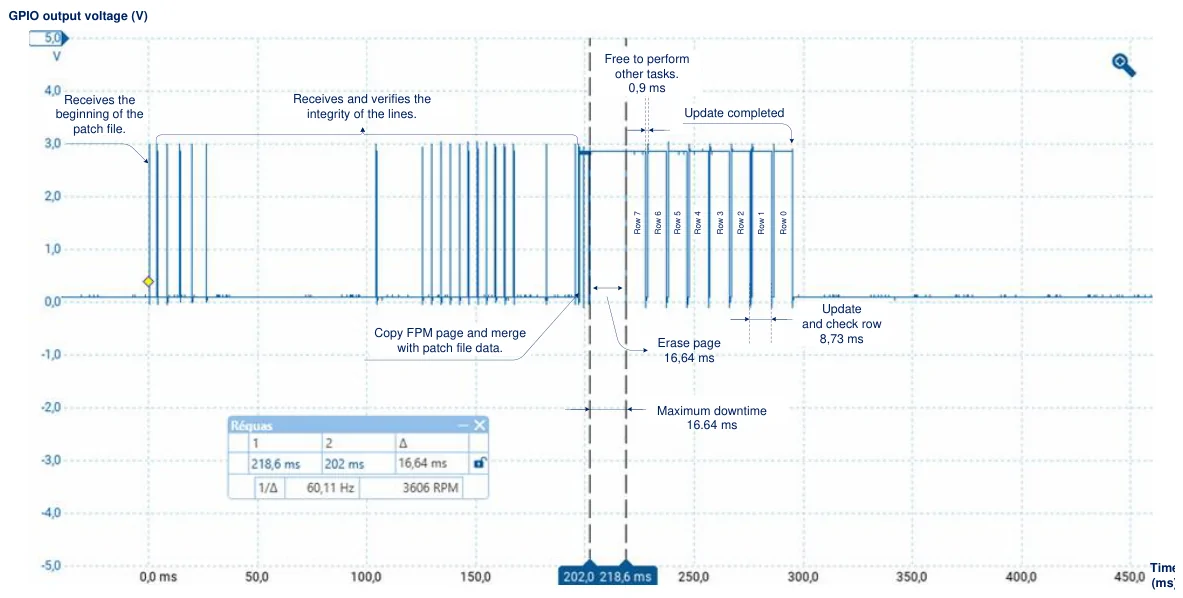
Timing diagram illustrating the firmware update process employing row-level FPM programming distributed across multiple RTS cycles.

Although the temporal behaviour remained similar to that observed for the application-level update strategy, a significant reduction in the volume of transferred data was achieved. For the same functional modification, the application-level update required the transmission of 2575 bytes, whereas the function-level update required only 911 bytes, corresponding to 35.38% of the originally transmitted volume. The third strategy analysed corresponds to the function-level update strategy employing row-level programming of the FPM. In this approach, the reprogramming process is distributed across multiple consecutive RTS cycles, with only a single row being programmed and verified during each scheduler iteration. The temporal behaviour of this approach is illustrated in Figure 11. The page erase time remained approximately constant relative to the previous strategy, with an average duration of 16.64 ms. However, the subsequent programming operations were distributed across multiple RTS cycles, resulting in an average programming time of approximately 8.73 ms per row.

Between consecutive programming operations, the system remained available to execute the remaining firmware tasks, significantly reducing the maximum continuous period of unavailability introduced during the update process. Experimental results showed that the longest continuous temporal blocking corresponded primarily to the time required to erase the target FPM page, averaging approximately 16 ms.

Although the row-level strategy reduces the maximum continuous CPU blocking interval, the affected application remains unavailable across multiple RTS cycles until all modified rows have been reprogrammed and validated. In the update scenario evaluated in this work, the total application unavailability was 93.68 ms for the row-level strategy, compared with 87.19 ms for the function-level strategy. This result illustrates the trade-off between reducing the continuous CPU blocking interval and the total duration of the affected application’s unavailability.

To preserve functional coherence during the partial update process, the FPM rows were reprogrammed in reverse order, starting from the highest-addressed row and ending with the row containing the application entry point. In addition, explicit invalidation of the Instruction Cache (I-cache) was performed after each programming operation, ensuring that the processor always executed the most recent version of the program memory.

It was also observed that this approach requires additional mechanisms to validate the functional dependencies between sub-applications. During the firmware update process, applications depending on functions that had not yet been fully reprogrammed could potentially attempt to execute FPM regions that were temporarily unavailable. To prevent this behaviour, the RTS was extended to explicitly validate the functional availability of applications prior to their execution (Figure 1).

To validate the robustness of the proposed mechanism, an experimental fault detection and recovery test was conducted during FPM updating. For this purpose, the data written to the program memory were deliberately corrupted after the programming operation in order to force the detection of inconsistencies during the update verification stage.

**Figure 12:**
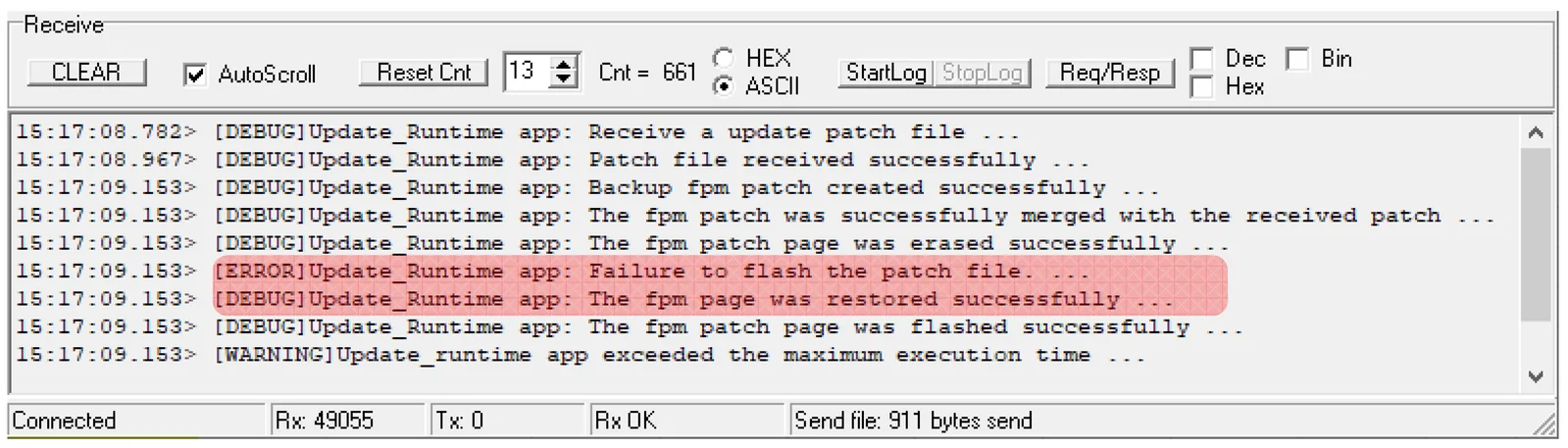
Logs generated by the report_debug sub-application during the firmware update process. Fault detection, and recovery are shown in the shaded area.

Figure 12 illustrates the logs generated by the report_debug sub-application during this experiment. The system successfully detected the discrepancy between the expected data and the data stored in the FPM, classifying the event as a critical failure.

Failures occurring during page erasure and row programming are handled by the update control and validation mechanisms, which prevent an incompletely updated region from being made available for execution again before successful verification. Similarly, the instruction cache invalidation stage is followed by execution-state synchronisation and validation mechanisms before the updated code is made available.

Following fault detection, the recovery mechanism automatically restored the FPM page that had been previously stored before the update process began. Throughout this procedure, the firmware remained operational and the program memory was successfully restored, thereby preventing firmware corruption from propagating to the rest of the system.

The different partial update strategies analysed exhibited distinct behaviours in terms of the temporal impact introduced during FPM reprogramming, the volume of transferred data, and the availability of applications during concurrent system execution.

Table 6 provides a comparative summary of the main experimental results obtained for the three implemented strategies, allowing the direct impact of reprogramming granularity on the temporal behaviour of the RTS and on the maximum continuous period of unavailability introduced during the update process to be observed.

The temporal statistical analysis includes 27 independent measurements for each operation. The results demonstrate low variability among the measurements (Table 7). The mean erase time ranged from 16.64 ms to 16.80 ms. The highest observed erase time was 17.03 ms for the function-level strategy. For the programming of a row (including verification of the row write operations), the mean time was 8.74 ms, with an observed maximum value of 8.90 ms. The P95 and P99 values confirm the low dispersion of the measured times, allowing the temporal variability of the memory operations to be characterised more comprehensively.

The experimental results demonstrated the feasibility of the proposed runtime partial Flash Program Memory update mechanism. The different strategies analysed revealed distinct trade-offs in terms of reprogramming granularity, the volume of transferred data, and the temporal impact introduced during the update process. It was further observed that functional memory segmentation, explicit application validation performed by the RTS, and cache coherence mechanisms play a decisive role in preserving the functional integrity of the system during runtime reprogramming operations.

In addition, the fault detection and recovery experiments confirmed the ability of the proposed mechanism to identify inconsistencies during critical programming operations and to automatically restore a consistent state of the program memory.

## 5. Discussion

The experimental results demonstrate the feasibility of the proposed mechanism on the PIC32MZ EF family, however, its fundamental principles are not limited to this specific architecture. The concept of runtime partial firmware updates was previously implemented and validated in microcontrollers with different architectures, including the Microchip PIC18F family, as demonstrated in previous work [1,2]. In that work, the update of specific firmware segments during execution on the PIC18F27K42 [23] was demonstrated, without requiring a system restart. The experience gained from those previous implementations constituted the basis for extending the concept to the PIC32MZ EF [22].

The previous validation on 8-bit microcontrollers made it possible to demonstrate the feasibility of the fundamental principle of partial updates during execution, whereas the migration to the PIC32MZ EF [22] introduced additional challenges associated with a higher-performance 32-bit architecture and the presence of an instruction cache. In this context, cache management and invalidation become necessary to ensure that, after Flash memory reprogramming, execution does not use previously stored and potentially outdated instructions.

Although manufacturers use different designations for the modules responsible for Flash memory management, such as NVM Controller, Flash Controller, or Flash Memory Controller, their fundamental function is equivalent: to provide controlled erase and programming operations for non-volatile memory by the application itself. Thus, the principle underlying the mechanism can be applied to other architectures that provide Flash programming mechanisms during execution. However, the complexity and efficiency of the implementation depend on the specific characteristics of each architecture, namely the memory organisation, the granularity of erase and programming operations, the Read-While-Write capability, and the presence of cache mechanisms.

In addition to the PIC18F family, architectures such as STM8L [28], MSP430F5529 [29], STM32F070 [30], and STM32H7 [31] were examined, taking into account the characteristics of their respective Flash memory controllers and the requirements of the proposed mechanism. This analysis makes it possible to verify that the principle of runtime partial firmware updates does not depend exclusively on the PIC32MZ EF architecture, although the specific characteristics of each platform, namely the memory organisation, the granularity of erase and programming operations, the Read-While-Write capability, and the presence of instruction cache mechanisms, determine different levels of adaptation and complexity.

The experimental results demonstrate that the mechanism maintains operational continuity during the update process and preserves a valid firmware state after the completion of each programming stage. This behaviour results from the combination of the segmentation of write operations, the synchronisation provided by the Runtime Scheduler, and the successive verification and validation stages performed before an updated application is made available, reducing the possibility of executing inconsistent or incomplete code.

Concurrent execution is therefore preserved selectively. Unaffected tasks continue to execute, while those whose code is located in the FPM region being updated are temporarily suspended to prevent the execution of erased or partially programmed code.

The demonstration used a deliberately simple functional modification. This allowed us to observe the outcome of the update directly and isolate the evaluation of the runtime reprogramming mechanism from the effects associated with the application’s functional complexity.

A statistical analysis of erase and programming times demonstrates minimal temporal variability in the memory operations. The maximum observed erase time was 17.03 ms, and the maximum observed programming time for a row was 8.90 ms. These values represent the worst case observed during the experiments and do not constitute a formal WCET bound.

The total duration of application unavailability may be affected by functional dependencies between tasks or sub-applications. Although the system provides mechanisms for callback and associated content validation before execution to prevent the activation of incomplete or inconsistent code, functional dependencies extending to memory regions that are temporarily unavailable may increase the temporal impact of update unavailability.

A critical scenario for any runtime Flash memory reprogramming mechanism remains the loss of power during the update process. Unlike other conditions that can be detected by the internal verification stages of the process, a power interruption may interrupt the reprogramming process before the affected memory region reaches a consistent state. This scenario, identified in previous work [1], was mitigated through the integration of a hardware-based energy backup mechanism, designed to ensure the completion of the programming operation. Additionally, fault injection tests confirmed the ability of the mechanism to detect and prevent the activation of inconsistent states during the update process.

Although the use of static and absolute function allocation provides deterministic addressing across different firmware versions, it requires a greater reservation of memory resources. To allow functions to evolve without altering the location of the remaining regions, a margin of approximately 25% above the number of rows required for each function was adopted. This additional capacity remains associated with the respective sub-application and can be used in future versions. This allows code modifications to be accommodated without reallocation of the remaining functions. This strategy therefore represents a trade-off between the predictability of the memory map and the efficient use of the available resources.

The magnitude of this overhead was evaluated by comparing the segmented and non-segmented configurations. The non-segmented configuration used 63,452 bytes of FPM and 3665 bytes of RAM, while the segmented configuration required 95,962 bytes of FPM and 6480 bytes of RAM. Segmentation therefore resulted in a 51.2% increase in FPM utilisation and a 76.8% increase in RAM utilisation. These increases resulted from the explicit reservation of independent regions for each module, the margins allocated for future expansion, and the organisation required to maintain deterministic addresses across firmware versions.

Table 8 summarises the Flash and RAM memory footprint of the Runtime Scheduler and the Runtime Update application in the proposed mechanism, distinguishing between the memory effectively used and the memory reserved for each of these components.

Although this strategy requires more memory reservation, it enables modifications to a single function to be carried out without the automatic reallocation of the remaining functions. This maintains stable firmware organisation across versions. This trade-off is particularly relevant for microcontrollers with limited memory resources and should be considered during the initial definition of the memory map, the size of the reserved regions, and the expansion margin allocated to each sub-application.

From a security perspective, the integrity verification used during the update process makes it possible to detect corruption or alterations in the received data, but it does not guarantee the authenticity of the firmware source or prevent unauthorised updates from being injected. The mechanism presented in this work therefore does not include cryptographic authentication mechanisms, digital signatures, rollback protection, or verification of an authorised firmware source. These mechanisms constitute a future extension, particularly relevant for integrating the mechanism with OTA infrastructures and for its use in distributed IoT and Cyber-Physical systems.

A direct quantitative comparison with existing runtime firmware update and hot-patching approaches is constrained by significant differences in the target platforms, memory organisations, update models, and experimental conditions [1,2,6,12,13,16,19,20]. The reported metrics, such as update time, unavailability duration, memory overhead, and update size, do not necessarily represent equivalent quantities across these different approaches.

Some approaches, for example, place the patch in RAM and redirect execution from the original code [12,13], while others use additional memory to accommodate the updated code [6] or apply differential or functional block-based updates via specific update procedures [16,19,20]. In contrast, the proposed mechanism involves the selective reprogramming of the program Flash memory during system execution, requiring explicit management of the affected memory region and preservation of the execution state.

Consequently, directly comparing these metrics numerically across different approaches could potentially be misleading. Therefore, the quantitative evaluation in this work is limited to the three update granularities implemented and evaluated under identical hardware, memory organisation, Runtime Scheduler, and experimental conditions. The related approaches are instead compared based on the characteristics objectively reported in the literature, including update models, the need for a reboot, memory requirements, update granularity, support for concurrent execution, and runtime behaviour [1,2,6,12,13,16,19,20].

## 6. Conclusions

This work presents the development and experimental validation of a runtime partial firmware update mechanism for multitasking embedded systems based on a 32-bit microcontroller architecture using the PIC32MZ2048EFM100 [22]. The proposed mechanism allows the selective reprogramming of FPM regions without restarting the system, integrating the update process with the Runtime Scheduler while maintaining the execution of unaffected tasks during reprogramming.

Three partial update strategies were implemented and experimentally evaluated at the application, function, and row levels, allowing the analysis of different trade-offs between update granularity, transmitted data volume, and temporal impact on the system. For the demonstration update used to validate the concept, the function-level strategy reduced the transmitted patch size to approximately 35% of that required for an equivalent application-level update. The row-level strategy reduced the maximum continuous CPU blocking interval to 17.03 ms by distributing the programming operations across multiple RTS cycles, although the affected application remained unavailable for 93.68 ms in the evaluated scenario.

The evaluation also demonstrated that executing partial updates on a PIC32MZ EF 32-bit microcontroller with an instruction cache (I-cache) requires explicit cache management and invalidation mechanisms, as well as validation of the state of the memory regions before the execution of the associated tasks. Integrating these operations with the RTS enabled the concurrent execution of unaffected tasks while the FPM regions under update remained temporarily unavailable.

The results further support the feasibility of the proposed runtime partial firmware update mechanism on high-performance microcontrollers such as the PIC32MZ EF family, highlighting the reduction in transmitted data volume through function-level updates and the reduced continuous temporal blocking achieved through row-level updates. The validation and recovery tests further confirmed the ability of the mechanism to detect abnormal conditions and prevent the execution of firmware regions in incomplete update states.

Future work will focus on automatic dependency management between applications, adaptive update strategies, security mechanisms for firmware distribution authentication and authorisation, and integration with remote OTA update infrastructures for distributed embedded and IoT/Cyber-Physical systems subject to advanced availability and fault-tolerance requirements.

## Figures and Tables

**Table 1:** Main memory and cache characteristics of the PIC32MZ2048EFM100 [22].

Description	Size	Units
Page Size	16,384	Byte
Row Size	2048	Byte
Erase Page Size	16,384	Byte
User Pages	128	Page
User Rows	1024	Row
I-Cache	16,384	Byte
D-Cache	4096	Byte

**Table 2:** Summary of the implemented update strategies.

Update Granularity	Continuous Temporal Blocking	Program Memory Inconsistency Window	Implementation Complexity
Page	High	Short	Low
Function	High	Moderate	Moderate
Row	Low	Long	High

*Note:* “Low”, “Moderate”, and “High” refer to the relative hardware and mechanism requirements needed to support the update approach, rather than implementation time, development effort, or software complexity.

**Table 3:** Distribution of the Sensor_Node task sub-application in Flash Program Memory.

Sub-Application	Page_ID	Row_ID
*Sensor_Node*	4	7
*Sensor_Node*	5	0–1

**Table 4:** Allocation of sub-applications across the rows of page 4 of the FPM.

Sub-Application	Page_ID	Row_ID
*analog_front_end*	4	0–1
*hardware_monitor*	4	2–3
*oscillators*	4	4–6
*report_debug*	4	7

**Table 5:** Program memory footprint associated with selected functions of the hardware_monitor sub-application after functional segmentation.

Symbol	Address	Row_ID	Size [Bytes]
hardware_monitor_set_task_status	0x9D011580	2	56
hardware_monitor_check	0x9D011600	2	436
hardware_monitor_initialize	0x9D011880	2	256

**Table 6:** Comparative analysis of the experimental results obtained for the implemented partial update strategies.

Parameter	Application-Level Update	Function-Level Update	Row-Level Update
Intel Hex File records	56	21	21
Intel Hex File size [Bytes]	2575	911	911
Updated bytes in the μC FPM	876	288	288
Average overall update process [ms]	434.90	258.74	266.64
Update and check page task [ms]	69.64	70.23	69.84
Erase page task [ms]	16.68	16.80	16.64
Maximum downtime [ms]	86.32	87.19	16.64

**Table 7:** Flash erase page and individual row-programming operation duration statistics.

Operation Time	Erase in Application-Level Update	Erase in Function-Level Update	Erase in Row-Level Update	Individual Row Programming
Mean time (ms)	16.68	16.80	16.64	8.74
Minimum time (ms)	16.46	16.57	16.43	8.60
Maximum time (ms)	16.97	17.03	16.86	8.90
P95 (ms)	16.93	16.98	16.80	8.90
P99 (ms)	16.97	17.02	16.84	8.90
Standard deviation (ms)	0.14	0.13	0.13	0.10

**Table 8:** Memory footprint of the Runtime Scheduler and Runtime Update application in the proposed mechanism.

Component	Flash Used (B)	Flash Reserved (B)	RAM Used (B)	RAM Reserved (B)
Runtime Scheduler (RTS)	3240	4080	684	896
Runtime Update Application	7644	12,272	33,416	33,791

*Note:* The reported Flash and RAM footprints were obtained from the linker map generated for the final compiled firmware.

## Data Availability

The original contributions presented in this study are included in the article/Supplementary Materials. Further inquiries can be directed to the corresponding author.

## Supplementary Materials

The following supporting information can be downloaded at https://www.mdpi.com/article/10.3390/s26185813/s1.

## Abbreviations

The following abbreviations are used in this manuscript:

D-cache	Data Cache
FPM	Flash Program Memory
I-cache	Instruction Cache
IoT	Internet of Things
MCU	Microcontroller Unit
NVM	Non-Volatile Memory
OTA	Over-The-Air
FOTA	Firmware-Over-The-Air
PIC	PIC microcontroller family
RTOS	Real-Time Operating System
RTS	Runtime Scheduler
